# The Flavonoid Biosynthesis and Regulation in *Brassica napus*: A Review

**DOI:** 10.3390/ijms24010357

**Published:** 2022-12-26

**Authors:** Yuan-Yuan Chen, Hai-Qin Lu, Kai-Xuan Jiang, Yi-Ran Wang, You-Ping Wang, Jin-Jin Jiang

**Affiliations:** 1 Jiangsu Provincial Key Laboratory of Crop Genetics and Physiology, Yangzhou University, Yangzhou 225009, China; 2 Joint International Research Laboratory of Agriculture and Agri-Product Safety, The Ministry of Education of China, Yangzhou University, Yangzhou 225009, China

**Keywords:** *Brassica napus*, flavonoid, plant development, stress response, regulation

## Abstract

*Brassica napus* is an important crop for edible oil, vegetables, biofuel, and animal food. It is also an ornamental crop for its various petal colors. Flavonoids are a group of secondary metabolites with antioxidant activities and medicinal values, and are important to plant pigmentation, disease resistance, and abiotic stress responses. The yellow seed coat, purple leaf and inflorescence, and colorful petals of *B. napus* have been bred for improved nutritional value, tourism and city ornamentation. The putative loci and genes regulating flavonoid biosynthesis in *B. napus* have been identified using germplasms with various seed, petal, leaf, and stem colors, or different flavonoid contents under stress conditions. This review introduces the advances of flavonoid profiling, biosynthesis, and regulation during development and stress responses of *B. napus*, and hopes to help with the breeding of *B. napus* with better quality, ornamental value, and stress resistances.

## 1. Introduction

Flavonoids are a group of secondary metabolites that broadly exist in plants and play important roles in plant development and stress responses by participating in various physiological processes [1,2]. Flavonoids are mainly compounds with C6-C3-C6 framework, including chalcones, flavans, flavones, flavonols, anthocyanins, and proanthocyanidins (PAs, or condensed tannins) that are synthesized through the flavonoid biosynthetic pathway [3]. Aside from these, stilbenes have also been reported as a group of flavonoids with C6-C2-C6 framework [4]. Hitherto, over 8000 flavonoids have been identified in plants [5]. As reported, flavonoids are major contributors for red, pink, blue, and purple pigmentations in plant organs (i.e., seeds, fruits, flowers, and leaves), and are also involved in plant adaptations to various abiotic and biotic stresses [6,7,8]. For example, the endoplasmic reticulum (ER)-derived flavonoids in *B. napus* anthers are mainly accumulated in tapetum cells to protect pollens from light damage and water stress [9]. Furthermore, these compounds are valuable to the quality of plant products, and have been intensively studied for their health and medical benefits against different diseases (i.e., cancers, cardiovascular diseases, and inflammatory diseases) [10,11,12,13].

Rapeseed (*Brassica napus* L.), as the second leading oil crop in the world, is a natural allotetraploid widely cultivated for its vegetable value, edible oil production, and animal foraging value [14]. Previously, breeding rapeseed with a yellow seed coat has been preferred for better oil and protein content, and less anti-nutrients (i.e., lignin, proanthocyanidins). Lately, rapeseed germplasms with colorful flowers have been bred for flower-based tourism and city ornamentation in China [15,16]. Moreover, rapeseed with purple leaves and inflorescence is rich in anthocyanins and provides valuable nutrients for human health [17]. These traits are closely related to flavonoid biosynthesis, and many research papers focusing on the gene screening and molecular regulation of flavonoid synthesis in rapeseed have been reported [18,19,20]. However, due to the genome complexity and narrow genetic background, the regulation mechanism of flavonoid biosynthesis in *B. napus* is very complicated and has not been fully elucidated yet. Furthermore, flavonoids have been predicted to have functions in stress responses of rapeseed, but no candidate gene or molecular regulatory network is available yet [21,22]. Here, we reviewed the research progress on flavonoid biosynthesis and molecular regulation mechanisms in *B. napus*, which should be valuable to rapeseed breeding in the future.

## 2. Flavonoid Biosynthesis in *Arabidopsis*

**Figure 1:**
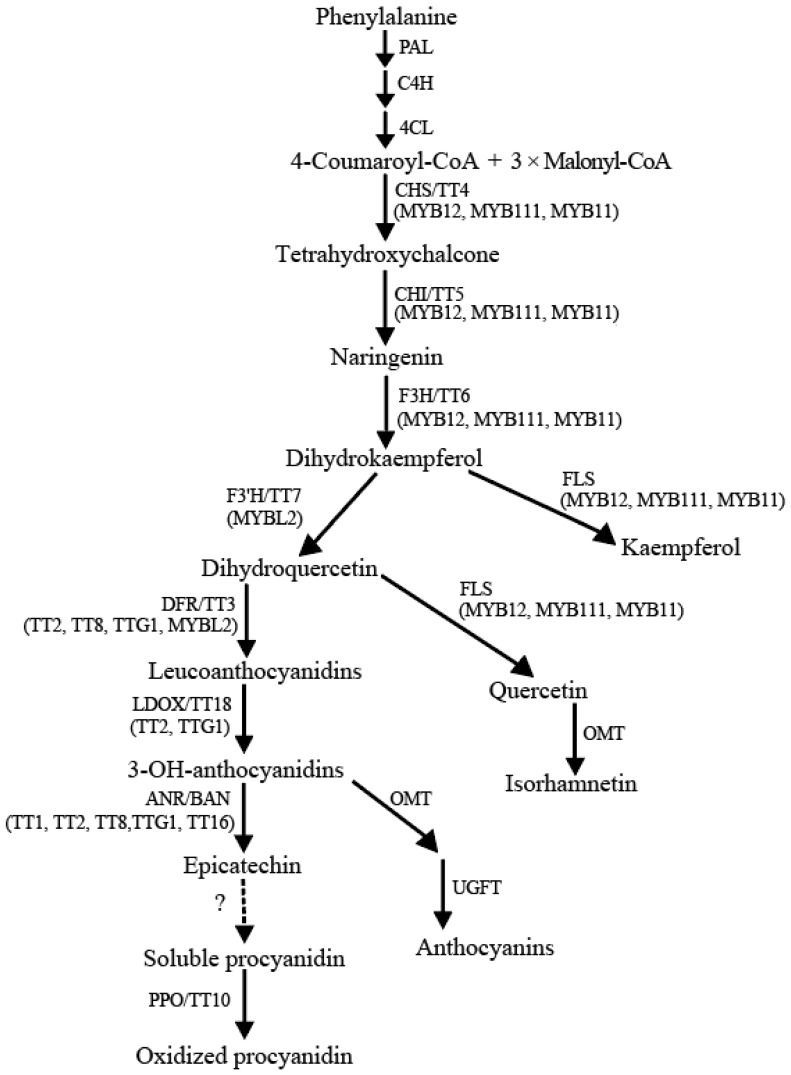
The major branch of flavonoid biosynthesis pathway in *Arabidopsis*.

Flavonoid biosynthesis and regulation are conserved in plants, which has been well characterized in *Arabidopsis* [2], cereal crops (i.e., rice, wheat) [23,24,25], oil crops (i.e., soybean, rapeseed) [26,27], flower plants (i.e., chrysanthemum, peony) [28,29], vegetables (i.e., cauliflower, radish) [30,31], and fruits (i.e., grape, apple) [32,33]. In *A. thaliana*, flavonols, anthocyanins, isoflavonols, and proanthocyanidins are the major types of flavonoids. Flavonols and PAs are only deposited in seeds [2]. Quercetin, kaempferol, and isorhamnetin are three groups of flavonols that could form flavonol derivatives, anthocyanins, or tannins. Catechin and epicatechin are two stereoisomers which form the polymers of PAs [34]. As reported, *transparent testa* (*TT*) gene mutation induced a light yellow seed coat in *A. thaliana*. These genes have been classified as early and late biosynthesis genes (EBGs and LBGs), and transcriptional regulatory factors in the flavonoid biosynthetic pathway [35]. The EBGs include *TT4/CHS*, *TT5/CHI*, *TT6/F3H*, and *TT7/F3′H*; the LBGs include *TT3/DFR*, *TT18/LDOX/ANS*, *BAN/ANR*, *TT12*, *TT19/GSTF12/GST26*, and *AHA10*. The MYB-bHLH-WD40 (MBW) complexes are important regulators in the flavonoid pathway, including TT2-TT8-TTG1, MYB5-TT8-TTG1, TT2-EGL3-TTG1, and TT2-GL3-TTG1 [36]. In addition, PAP1/MYB75 and PAP2/MYB90 were also identified as components of the MBW complexes in *A. thaliana* (Figure 1) [37].

## 3. Flavonoid Profiles Tentatively Identified in Rapeseed

In *B. napus*, the flavonoid profiles in seeds, roots, leaves, and petals have been reported. Farag et al. (2013) identified 68 phytochemical compounds in different organs (root, leaf, stem, inflorescence, and seed) of *B. napus* via UPLC-QTOF-MS, including 13 hydroxycinnamic acids/sinapoyl cholines, 25 flavonols, and one flavanone [38]. Shao et al. (2014) reported a comprehensive profile of flavonoids and hydroxycinnamic acid derivatives in black seeds of *B. napus*, including 39 kaempferols, 11 isorhamnetins, 5 quercetins, 6 flavanols, and 30 hydroxycinnamic acid derivatives [39]. Qu et al. (2013) identified 35 flavonols in the seed coat of *B. napus*, including 13 and 2 compounds specific to yellow and black seeds, respectively. They found the flavonoid biosynthetic genes, anthocyanin- and proanthocyanidin-specific genes, as well as regulators in the flavonoid synthetic pathway were repressed in yellow seeds compared with that in black seeds [40]. Li et al. (2009) created yellow-seeded rapeseed germplasms from *B. napus-Sinapis alba* somatic hybrids, and Jiang et al. (2013) revealed the 16 flavonoids differentially accumulated between yellow and black seeds, which might be due to the repressed flavonoid biosynthetic genes in yellow seeds compared with black seeds [41,42]. Later, Wang et al. (2018) compared the content of 56 phenolic components between yellow- and black-seeded *B. napus*, including 15 hydroxycinnamic acid derivatives, 21 kaempferols, 10 isorhamnetins, 5 quercetins, 5 epicatechins and derivatives, which might be correlated with the seed coat color variation in the progeny of *B. napus-S. alba* hybrids [43]. Yin et al. (2019) identified 15 phenolic acids and 28 flavonoids in rapeseed accessions with pale white, yellow, pink, and red petals, which might be responsible for the petal color variation [44]. Recently, 39 flavonoids including 35 anthocyanins were identified in three rapeseed cultivars with purple, reddish-green, and green leaves. A total of 22 anthocyanins that exhibited different accumulation might contribute to the leaf color variations, including cyanidin and kaempferol derivates. The differentially expressed genes among three rapeseed cultivars were most enriched in metabolic pathways (i.e., flavonoid biosynthesis, secondary metabolite biosynthesis). Many regulatory genes (i.e., *MYB*, *ERF*, *NAC*, and *bHLH*) and structural genes (i.e., *BnDFR*, *BnANS*, *BnUGTs*, and *BnGSTs*) related to anthocyanin biosynthesis were up-regulated in purple and reddish-green leaves more than that in green leaves [17]. The flavonoid profiles have also been reported in rapeseed under different growth conditions. Shen et al. (2022) comprehensively analyzed the metabolic profiles in roots and leaves of rapeseed grown under normal nitrogen (HN) and nitrogen-deficient (LN) conditions. They identified that three anthocyanins and 22 flavonols were increased, while one anthocyanin, three flavonols, and five flavanones were decreased in leaves under LN compared to HN conditions. In roots of rapeseed, 13 flavonoids were increased and 12 were decreased in plants grown under LN compared to HN conditions [45]. Although many flavonoids have been characterized in different organs of rapeseed and stressed plants, the regulation of flavonoid accumulation during rapeseed development and in response to various stresses has not been fully elucidated. Hitherto, only a few genes and loci related to flavonoid regulation in *B. napus* have been reported (Table 1).

## 4. Regulation of Flavonoid Biosynthesis in Seeds of *B. napus*

Flavonoid accumulation could affect seed coat color, seed germination, and seed meal quality of *B. napus*. Jia et al. (2012) proved that increased proanthocyanidin content inhibited seed germination of *Arabidopsis* and *B. napus*, which was due to the ABA accumulation in seeds. Proanthocyanidin might play a role as a doorkeeper during seed germination [61]. Through a genome-wide association study (GWAS) of 96 *B. napus* accessions, Bhinder et al. (2022) identified 789 significant single nucleotide polymorphisms (SNPs) associated with seed meal quality of rapeseed. Three candidate genes, *SOT12*, *SK1*, and *UGT88A1*, related to flavonoid biosynthesis were identified, which should be helpful for reducing anti-nutritional components and improving seed meal quality of *B. napus* [62]. In *B. napus*, many studies of flavonoid biosynthesis and regulation related to seed coat color have been reported.

### 4.1. Gene Expressional Changes in Yellow Seed of B. napus

Yellow seed is a quantitative trait preferred by breeders for its advantages of high oil and protein content and less anti-nutritional components (i.e., lignin, fiber, pigments, and polyphenols) compared with its black-seeded counterpart [63]. However, due to the genome complexity of rapeseed and genetic background of yellow-seeded germplasms, yellow seed trait (YST) is sensitive to harvesting time, temperature and fertilizers. The related molecular mechanisms of this phenotype and its connection with other seed traits still need to be elucidated. As reported, *F3′H*, *TT1*, *TT2*, *PAL*, *BAN*, *TTG1*, and *TT10* have been cloned and proven to have functions in flavonoid biosynthesis [46,47,64,65,66,67,68,69]. Yu (2013) reviewed the molecular mechanism of manipulating seed coat color in *Brassica* species, including the homologous *TTs* cloned in *Brassicas* [63]. Homologous *TT* genes related to seed coat variation in Brassicaceae have also been analyzed, including 95 copies of 21 *TTs* in *B. napus* [18]. Liu et al. (2016) identified all the proanthocyanidin-associated genes in the genome of *Brassicas*, including the 58 homologous genes in *B. napus* [70]. Based on the transcriptome analysis of yellow- and brown-seeded near-isogenic lines (NILs) of *B. napus*, Hong et al. (2017) found that the down-regulated genes in yellow seed coats were enriched in phenylpropanoid and flavonoid biosynthesis [71]. Jiang et al. (2019) compared the gene expression in developing seeds of yellow seeds from *B. napus*-*Sinapis alba* somatic hybrids with black rapeseeds, and found many differentially expressed genes (DEGs) were involved in flavonoid and phenylpropanoid biosynthesis, phenylalanine metabolism, flavone and flavonol biosynthesis, fatty acid (FA) biosynthesis, and metabolism [72]. Lin et al. (2020) analyzed the novel transcripts in yellow- and black-seeded rapeseeds, and identified the alternative splicing profiles at different seed developmental stages. Finally, they screened 24 differentially alternative splicing (DAS) genes (i.e., *BAN*, *CHI*, *DFR*, *AHA10*, *STK*, *TT5/8/10/12/16*) that might be related to the seed coat color variation, and would be valuable to yellow seed breeding [73]. These DEGs would be helpful for the molecular dissection of yellow seed trait in rapeseed.

### 4.2. Quantitative Trait Loci of Yellow Seed Trait in B. napus

Hitherto, many QTLs related to the yellow seed trait of *B. napus* have been reported. Badani et al. (2006) identified a major QTL of seed color on C08, which was co-located with a major QTL of acid detergent fiber (ADF) and confirmed by Zhang et al. (2011) afterwards [74,75]. Similarly, a major QTL on A09 was reported to control seed color and fiber content [76,77]. The locus reported by Liu et al. (2012) was tightly linked to a major QTL for seed acid-detergent lignin (ADL) content, and *BnaA.CCR1.A9* with a functional mutation in the first exon might be the candidate gene for yellow seed with reduced ADL content [76]. Afterwards, they reported 11 QTLs for seed color and fiber traits across four different environments, and constructed a high-density map with considerably improved QTL resolution [78]. Using the recombinant inbred lines (RILs) between *B. napus* cv. Zhongyou 821 and yellow seed line GH06, Qu et al. (2017) constructed a linkage map with 1089 markers, identified 72 eQTLs associated with the 18 flavonoid biosynthetic genes, and found *bZIP25*, *MYC1*, *MYB51*, and *MYB52* were candidate genes in the eQTL hotspots [79]. Wang et al. (2017) also identified two homologous loci controlling seed coat color on C08 and A09 through GWAS analysis [80]. In addition, many minor QTLs of YST were also detected on C02, C05, C06, C07, A01, A04, A07, and A08 [80,81]. Recently, Chao et al. (2022) identified a major yellow-seed QTL, *cqSC-A09*, and found the advantageous allele significantly increased the oil content, and reduced the pigment and fiber content in seeds of *B. napus*. Among the 648 genes in *cqSC-A09*, *BnaA09.JAZ1*, *BnaA09.GH3.3*, and *BnaA09.LOX3* were predicted as major candidates after sequence variation annotation, expression differences and interaction network analysis [82].

### 4.3. Functionally Characterized Genes Regulating Yellow Seed Trait of B. napus

Although many QTLs and DEGs related to YST have been identified in *B. napus*, only a few genes regulating flavonoid biosynthesis and seed coat color of *B. napus* have been proven. Zhai et al. (2020) firstly created yellow-seeded *B. napus* through the CRISPR/Cas9 technique, and confirmed that homozygous mutation of *BnTT8* suppressed the expression of phenylpropanoid and flavonoid biosynthetic genes, inhibited proanthocyanidin accumulation in seed coat, improved seed oil and protein content, and altered FA composition of rapeseed [20]. Xie et al. (2020) also found that mutation of *BnTT2*, another regulator in flavonoid biosynthetic pathway, reduced flavonoids and improved FA composition in rapeseed [19]. Previously, RNAi of *BnTT1* and *BnTT10* revealed their function in regulating PA metabolism, lignin synthesis, seed coat pigmentation, and FA biosynthesis [46,47]. All these genes are homologs of *Arabidopsis TTs* in rapeseed, and none of the reported DEGs or candidate genes in the aforementioned QTLs have been functionally characterized.

## 5. Regulation of Anthocyanin Biosynthesis in *B. napus* Flowers

Apart from carotenoids, flavonoids such as chalcones, certain flavonols, and anthocyanins are also flower pigments, of which anthocyanins are major contributors for orange, pink, red, purple, and blue flowers [83,84]. In *Arabidopsis*, anthocyanin biosynthesis is regulated by MBW complexes consisting of a MYB protein (i.e., AtMYB113/114, MYBL2, AtPAP1/2), a bHLH protein (i.e., GL3, EGL3, TT8), and a WD40 protein (i.e., TTG1) [85]. Hitherto, only a few studies on anthocyanin-related rapeseed petal color have been reported. Overexpression of *Orychophragmus violaceus OvPAP2*, a homolog of *AtPAP2*, resulted in purple and red pigmentation on flower organs of *Arabidopsis* and rapeseed, respectively [48]. Based on the map-based cloning and gene functional analysis, Liu et al. (2020) proved that *BnaA09.ZEP* and *BnaC09.ZEP* negatively regulate the orange color in rapeseed petals by affecting the carotenoid content. Moreover, the expression of flavonoid biosynthetic genes and flavonoid profiles were also changed between the *BnaA09.ZEP/BnaC09.ZEP* mutants and their complementary lines [15]. Metabolomic and RNA-seq analysis on two developmental stages of unopened red, white, and yellow petals of rapeseed revealed that enriched flavonoids and up-regulated anthocyanin biosynthetic genes (i.e., *DFR*, *ANS*, and *UF3GT*) might be responsible for the red pigmentation. Repression of *BnaA03.ANS* in red-petal rapeseed changed the petal color to beige red and zinc yellow, while overexpression of *BnaA03.ANS* did not change the color of yellow petals [16]. Ye et al. (2022) found *BnaA07.PAP2*, *BnaMYBL2*, *BnaTT8* and structural genes (i.e., *DFR*, *ANS*, *UFGT* and *GST*) in anthocyanin biosynthesis were up-regulated in apricot and pink flowers compared with yellow and white flowers of rapeseed. They confirmed *BnaA07.PAP2* as a key gene regulating anthocyanin pigmentation of rapeseed flowers through map-based cloning and multi-omics analysis. Moreover, two insertions in −184 and −371 bp were responsible for the transcriptional activation of *BnaA07.PAP2* and anthocyanin-related genes. Introducing *BnaA07.PAP2^In−184−317^* into rapeseed cultivar ‘Westar’ changed the yellow petals to apricot petals [49]. Mutation of the carotenoid isomerase gene *BnaCRTISO* altered the gene expression in flavonoid biosynthesis, carotenes and xanthophylls synthesis pathway, reduced chalcone content and increased carotene content, thus inducing white petals and yellowish leaves in rapeseed [50].

## 6. Flavonoid Regulation in Rapeseed Leaves

As mentioned above, anthocyanins are helpful to human health for their antioxidant, chemoprotective, and anti-inflammatory properties. *B. napus* germplasms with increased anthocyanin content have been used as vegetables with better nutritional values. Mapping and gene identification of red and purple *Brassicas* were mainly reported in *B. rapa*, *B. juncea*, and *B. oleracea* [86,87,88,89]. Due to the complicated genome divergence and homologous genes of *B. napus*, the molecular mechanism of genes controlling the purple leaf phenotype has not been fully elucidated. Only a few genes have been reported with functions in regulating anthocyanin biosynthesis in rapeseed leaves.

Overexpression of *Arabidopsis PAP1* stimulated flavonoid and phenolic biosynthetic gene expression in *B. napus*, thus promoting cyanidin, pelargonidin, quercetin, sinapic acid content, and inducing purple leaves and stems in the transgenic plants [54]. Ectopic expression of *BnGL3-1*, which encoded a nucleus-located transcription factor, increased the trichome number and anthocyanin accumulation in true leaves of *Arabidopsis gl3-3* mutant by manipulating the expression level of genes involved in trichome formation (i.e., *GL2*, *MYB23*) and anthocyanin biosynthesis (i.e., *PAP1*, *LBD37/38*, *F3H*, *F3′H*, *DFR*, *ANS*) [53]. Goswami et al. (2018) created a resynthesized *B. napus* line Rs035 with high cyanidins and malvidins by crossing *B. rapa* and *B. oleracea* with high anthocyanin content. Three EBGs (*CHS*, *CHI*, and *F3H*), four LBGs (*FLS1*, *DFR*, *ANS*, and *UGT75C1*), two regulatory genes (*MYB111* and *TT8*), and a transporter gene (*TT19*) were up-regulated in Rs035 than in the diploid parents, which might be putative candidates for the enhanced anthocyanin content in Rs035 [90]. Chen et al. (2020) identified three alternatively spliced isoforms of *BnaPAP2.A7* in a rapeseed introgression line from the progenies of (*B. rapa* × *O. violaceus*) × *B. napus*, which exhibited purple leaves and petioles. Only the *BnaPAP2.A7-744* isoform promoted anthocyanin accumulation, and changed the green leaves to purple leaves through up-regulation of EBGs, LBGs and transcription factors in the flavonoid biosynthetic pathway, while overexpression of *BnaPAP2.A7-910* or *BnaPAP2.A7-395* repressed anthocyanin-related genes, and could not change the leaf color [52]. He et al. (2021) analyzed the expression pattern of anthocyanin biosynthetic genes in two rapeseed lines with purple and green leaves, and their F1 progenies. They found the up-regulation of *TT8*, *DFR*, *ANS*, *UFGT*, and *TT19* promoted anthocyanin biosynthesis in early development of purple leaves, but these genes were down-regulated and anthocyanin content was decreased at the late developmental stage [91]. Recently, five anthocyanins (mainly cyanidins) were identified in the leaves and stem bark of a purple rapeseed line PR01. A total of 157 anthocyanin biosynthesis genes in rapeseed were screened through comparative analysis with *Arabidopsis* genes, among which *BnaA07.PAP2* and *BnaC06.PAP2* were identified as the key regulators of anthocyanin biosynthetic genes (i.e., *DFR*, *F3′H*, and *ANS*) and high anthocyanin content in PR01 [27]. Schilbert et al. (2021) characterized the gene structure, genomic location, and expression pattern of 13 *FLS* genes in rapeseed. They found the *BnFLS1-1* and *BnFLS1-2* could restore the flavonoid (i.e., kaempferol and quercetin derivatives) contents in *Arabidopsis ans/fls1* and *f3h* mutants, indicating the FLSs play a role as bifunctional enzymes in generating dihydrokaempferol and kaempferol [60]. Presently, only one locus *BnaA.PL1* on chromosome A03 was mapped for an anthocyanin-rich mutant of rapeseed, and *BnAPR2* was predicted to be a main candidate gene that encoded adenosine 5′-phosphosulfate reductase [51].

## 7. Flavonoid Regulation Related to Abiotic and Biotic Stress Responses in Rapeseed

Abiotic stresses, such as salinity, drought, cold, and logging, inhibit rapeseed growth and yield, and affect the seed quality, thus leading to great economic loss and restricting the worldwide cultivation area of *B. napus*. Flavonoids have been reported to have functions in plant response and adaptation to various environmental stresses. Moreover, flavonoids are also helpful for improving plant resistance to different biotic stresses. However, the molecular mechanism of how flavonoid biosynthesis is involved in plant response to different stresses has barely been reported.

### 7.1. Flavonoid Regulation in Response to Abiotic Stresses

Ellerstrom et al. (2005) found overexpression of *BnET*, a gibberellin-response repressor, promoted anthocyanin accumulation in *Arabidopsis* and might be favorable to balance ABA and GA in plants [59]. Yu et al. (2014) analyzed the gene expression in siliques of rapeseed under heat stress, and found many genes in silique wall metabolic pathways and flavonoid synthesis (i.e., *PAL1*, *CHI*, *FLS1*, and *GST*) were down-regulated [92]. Anthocyanin content could be induced in rapeseed seedlings under sucrose, ABA, NaCl, and mannitol stress, but not under GA_3_ treatment. In the root tip of *B. napus*, flavonols were increased under sucrose, GA_3_, NaCl, and mannitol treatments, but not under ABA treatment. *BnFLS*, an early biosynthesis gene in the flavonoid biosynthetic pathway, could be induced by mannitol treatment, and inhibited by sucrose, GA_3_, and NaCl treatments. Overexpression of *BnFLS* recovered the flavonol content, and increased the reactive oxygen species (ROS) scavenging activity in an *Arabidopsis atfls1-ko* mutant [58]. Koeslin-Findeklee et al. (2015) analyzed the gene expression of different *B. napus* cultivars under nitrogen starvation, leaf shading, and detaching to screen the genes’ response to leaf senescence. They found genes related to flavonoid synthesis were induced by nitrogen starvation and leaf detaching, which would contribute to protect leaves against photooxidative stress [22]. Physiologic and transcriptomic analysis revealed that with high anthocyanin accumulation in rapeseed under salinity stress, the genes in anthocyanin biosynthesis were up-regulated (i.e., *CHS*, *DFR*, *PAP1*, *PAP2*, *bHLH122*, and *WD40*), which might be helpful for protecting leaves from stress-induced damage [93]. Overexpression of *Arabidopsis AtDFR* increased anthocyanin accumulation and improved salt tolerance of *B. napus* [57]. Based on the proteomic analysis of two *B. napus* cultivars with different cold tolerance, Mi et al. (2021) identified 34 common different abundant proteins (DAPs) between two cultivars that had been grown at −4 °C for 12 h and 24 h, including a candidate protein FLS that is involved in flavonoid biosynthesis and might be related to the ROS scavenging under cold stress [94]. Sami et al. (2020) revealed that melatonin significantly enhanced the anthocyanin content in rapeseed seedlings under cadmium or aluminum stresses, which might be helpful to the metal tolerance of *B. napus* [95]. The morphological and physiological parameters of two rapeseed germplasms with a green stem and a purple stem indicated the main function of anthocyanin accumulation in rapeseed under drought stress was to improve antioxidant capability and stress tolerance. Furthermore, the genotype with a purple stem exhibited higher drought tolerance than the green stem genotype, which might be related to the higher expression of anthocyanin biosynthetic genes and antioxidant enzymes [96].

### 7.2. Flavonoid Regulation in Response to Light Conditions

Aside from the aforementioned abiotic stresses, flavonoid accumulation was also changed in rapeseeds under different light conditions. Rapeseed in the main cultivation area of China, the Qinghai-Tibetan plateau, usually suffers light stress, but the mechanisms underlying rapeseed adaptation to light stress are still unknown. Gerhardt et al. (2008) found flavonoid accumulation in *B. napus* was suppressed by far-red light, while addition of UV-B altered the flavonoid composition [97]. Luo et al. (2021) found the anthocyanin (i.e., cyanidin, delphinidin, and petunidin) contents in rapeseed under high light stress were significantly increased compared to that grown under normal light conditions, and the genes in the anthocyanin biosynthetic pathway (i.e., *BnDFR*, *BnANS*, *BnPAP1*, and *BnGL3*) and the jasmonic acid biosynthetic pathway were also induced by high light stress [98]. Moreover, Groenbaek et al. (2019) found the annual or biennial life cycle and seasonal differences, other than developmental stages, had major effects on the levels of flavonoid glycosides and hydroxycinnamic acids in baby leaf rapeseed [99]. Flavonol and anthocyanin content in microgreens of *B. napus* and *B. juncea* were changed under different blue–red light ratios, which could be used as growth parameters in evaluating the nutritional values of microgreens [100]. UV-B irradiation could enhance the phenylpropanoid, flavonoid, and anthocyanin accumulation in *B. napus* seedlings by affecting the gene expression in secondary metabolite biosynthesis [101]. BnCRY1 and BnCRY2a, two homologs of *Arabidopsis* UV-B photoreceptors AtCRY1 and AtCRY2, were involved in regulating photomorphogenesis and anthocyanin accumulation of rapeseed seedlings. Overexpression of *BnCRY1* and *BnCRY2a* inhibited hypocotyl length and increased anthocyanin content in *B. napus* seedlings grown under blue and white light by affecting the gene expression associated with phytohormone synthesis and signaling, as well as cell wall components [55,56,102].

### 7.3. Flavonoid Regulation in Response to Biotic Stresses

Presently, only a few research papers on flavonoids involved in biotic stress responses have reported on *B. napus*. A kaempferol derivative, kaempferol 3-*O*-sinapoyl-sophoroside 7-*O*-glucoside (KSSG), has been identified as a flavonoid related to the improved resistance to cabbage seedpod weevil of the BC2 double haploid lines from *B. napus-S. alba* intergeneric hybrids. A QTL on linkage group N7 that explained 9.5% of the KSSG variation was identified [103]. Li et al. (2015) found that BnaMAPKKK4 induced ROS, malondialdehyde (MDA), and anthocyanin accumulation by interacting with BnaMPK3 to regulate ROS-induced cell death in tobacco leaves [104]. Islam et al. (2019) reported that cell wall-bound phenolic metabolites were significantly increased in resistant rapeseed cultivar after inoculation of *Xanthomonas campetris* pv. *campestris*, accompanied by increased jasmonic acid content and up-regulation of genes in the phenylpropanoid biosynthetic pathway [105]. An *anthocyanin-more* (*am*) mutant of rapeseed cultivar ‘Zhongshuang11’ was identified with improved *Sclerotinia sclerotiorum* resistance and waterlogging tolerance, which might be due to the higher anthocyanin content, and expressional changes of anthocyanin-related structural and regulatory genes in the mutant [106,107].

## 8. Conclusions and Perspectives

The flavonoid profiles in different organs, especially seeds, leaves, and petals of rapeseed, as well as *B. napus* seedlings under various stresses have been reported. This research clarified the specific compounds presented in different tissues, and the effects of environmental conditions on the accumulation pattern of flavonoids in *B. napus*, which would be helpful for studying the flavonoid regulation related to rapeseed development and stress responses. The molecular regulation of flavonoid biosynthesis in *A. thaliana* has been well characterized, but the genes involved in flavonoid biosynthesis in *B. napus* were not fully identified, mainly due to the genome polyploidization that made the flavonoid regulation more complex in rapeseed. Presently, many genes related to flavonoid biosynthesis in *B. napus* have been screened through transcriptional comparison between germplasms exhibiting different flavonoid content in the seed, petal, leaf, or stem, or under different growth conditions. Only a few QTLs of flavonoid biosynthesis in seed coats, leaves, and petals have been identified. These genes are valuable for the dissection of the regulatory network of flavonoid synthesis and the biological roles of these chemicals in plant adaptation. However, the functions of most candidate genes regulating flavonoid synthesis were not analyzed. Most of the characterized genes were orthologous of *Arabidopsis TT* genes, such as *TT1/2/3/8/10*, *FLS*, *ANS*, and *PAP1/2*. Here, we summarized the research on flavonoid biosynthesis and regulation in *B. napus*, and hope these metabolic profiles and candidate genes will facilitate the quality improvement, ornamental value, and stress resistances of rapeseed in the near future.

## Figures and Tables

**Table 1:** The functionally characterized genes regulating flavonoid synthesis in *B. napus*.

Gene	Origin	Function	Phenotype	Reference
*BnTT8*	*Brassica napus*	*BnTT8* mutation suppressed the phenylpropanoid and flavonoid biosynthetic gene expression, and inhibited proanthocyanidin accumulation in seed coat of rapeseed.	Seed color	[20]
*BnTT1*	*Brassica napus*	Silencing of *BnTT1* reduced flavonoid accumulation and fatty acid biosynthesis through altering gene expression in flavonoid and fatty acid biosynthesis.	Seed color	[46]
*BnTT10*	*Brassica napus*	Silencing of *BnTT10* increased soluable proanthocyanidins, decreased extractable lignin, and retarded pigmentation in seed coat of *B. napus*.	Seed color	[47]
*BnTT2*	*Brassica napus*	Mutation of *BnTT2* reduced flavonoids and improved fatty acid composition in seed of *B. napus*.	Seed color	[19]
*OvPAP2*	*Orychophragmus violaceus*	Ectopic expression of *OvPAP2* led to red anthers and petals in *B. napus*.	Petal color	[48]
*BnaA03.ANS*	*Brassica napus*	RNA interference of *BnaA03.ANS* repressed anthocyanin accumulation in red petal rapeseed.	Petal color	[16]
*BnaA07.PAP2*	*Brassica napus*	The insertions in −184 and −371 bp were responsible for the transcriptional activation of *BnaA07.PAP2* and anthocyanin-related genes, and resulted apricot petal color in rapeseed.	Petal color	[49]
*BnaA09.ZEP/BnaC09.ZEP*	*Brassica napus*	*BnaA09.ZEP* and *BnaC09.ZEP* negatively regulated the orange color in rapeseed petals by affecting the carotenoid and flavonoid content, as well as the expression of carotenoid and flavonoid biosynthetic genes.	Petal color	[15]
*BnaCRTISO*	*Brassica napus*	*BnaCRTISO* mutation reduced chalcone content and increased carotene content, thus changing the petal and leaf color of rapeseed.	Petal/leaf color	[50]
*BnaA.PL1*	*Brassica napus*	A QTL locus for anthocyanin-rich mutant of rapeseed, including a candidate gene *BnAPR2* that encoded adenosine 5’-phosphosulfate reductase.	Leaf color	[51]
*BnaPAP2.A7*	*Brassica napus*	Three isoforms of *BnaPAP2.A*7 identified in rapeseed introgression line were confirmed with different roles in manuplating anthocyanin accumulation in leaves.	Leaf color	[52]
*BnGL3-1*	*Brassica napus*	Ectopic expression of *BnGL3-1* increased the trichome number and anthocyanin accumulation in true leaves of *Arabidopsis gl3-3* mutant.	Leaf color	[53]
*AtPAP1*	*Arabidopsis thaliana*	Overexpression of *Arabidopsis PAP1* increased flavonoid and sinapic acid accumulation in leaves and stems of rapeseed.	Leaf/stem color	[54]
*BnCRY1/BnCRY2*	*Brassica napus*	Overexpression of *BnCRY1* and *BnCRY2a* increased anthocyanin content and regulated seedling photomorphogenesis of *B. napus*.	Seedling development	[55,56]
*AtDFR*	*Arabidopsis thaliana*	Overexpression of *Arabidopsis AtDFR* increased anthocyanin accumulation and improved salt tolerance of *B. napus*.	Salt tolerance	[57]
*BnFLS*	*Brassica napus*	Overexpression of *BnFLS* recovered the flavonol content in *Arabidopsis atfls1-ko* mutant.	--	[58]
*BnET*	*Brassica napus*	Overexpression of *BnET* promoted anthocyanin accumulation in *Arabidopsis*.	--	[59]
*BnFLS1-1/1-2*	*Brassica napus*	*BnFLS1-1* and *BnFLS1-2* restored the flavonoid content in *Arabidopsis ans/fls1* and *f3h* mutants.	--	[60]

## Data Availability

Not applicable.
